# Milk Production, Body Weight, Body Condition Score, Activity, and Rumination of Organic Dairy Cattle Grazing Two Different Pasture Systems Incorporating Cool- and Warm-Season Forages

**DOI:** 10.3390/ani11020264

**Published:** 2021-01-21

**Authors:** Kathryn E. Ritz, Bradley J. Heins, Roger D. Moon, Craig C. Sheaffer, Sharon L. Weyers

**Affiliations:** 1 West Central Research and Outreach Center, University of Minnesota, Morris, MN 56267, USA; ruhxx011@umn.edu; 2 Department of Entomology, University of Minnesota, St. Paul, MN 55108, USA; rdmoon@umn.edu; 3 Department of Agronomy and Plant Genetics, University of Minnesota, St. Paul, MN 55108, USA; sheaf001@umn.edu; 4 USDA North Central Soil Conservation Research Lab, Morris, MN 56267, USA; sharon.weyers@usda.gov

**Keywords:** grazing, teff, sorghum-sudangrass, organic dairy cows

## Abstract

**Simple Summary:**

Organic dairy cows were used to evaluate the effect of two pasture production systems on milk, fat, and protein production, somatic cell score, milk urea nitrogen, body weight, body condition score, and activity and rumination. Milk production increased when cows grazed sorghum-sudangrass compared to when they grazed perennial grasses and legumes. Warm-season annual grasses may be incorporated into grazing systems for organic dairy cattle while maintaining milk production and components.

**Abstract:**

Organic dairy cows were used to evaluate the effect of two organic pasture production systems (temperate grass species and warm-season annual grasses and cool-season annuals compared with temperate grasses only) across two grazing seasons (May to October of 2014 and 2015) on milk production, milk components (fat, protein, milk urea nitrogen (MUN), somatic cell score (SCS)), body weight, body condition score (BCS), and activity and rumination (min/day). Cows were assigned to two pasture systems across the grazing season at an organic research dairy in Morris, Minnesota. Pasture System 1 was cool-season perennials (CSP) and Pasture System 2 was a combination of System 1 and warm-season grasses and cool-season annuals. System 1 and System 2 cows had similar milk production (14.7 and 14.8 kg d^−1^), fat percentage (3.92% vs. 3.80%), protein percentage (3.21% vs. 3.17%), MUN (12.5 and 11.5 mg dL^−1^), and SCS (4.05 and 4.07), respectively. Cows in System 1 had greater daily rumination (530 min/day) compared to cows in System 2 (470 min/day). In summary, warm-season annual grasses may be incorporated into grazing systems for pastured dairy cattle.

## 1. Introduction

There has been an increased demand for organic dairy products due to interest in regenerative agriculture and grazing, as well as animal welfare concerns associated with dairy production systems [1]. Therefore, this demaand has increased the number of organic dairy farms in the United States. Pasturgre-based dairy production may be a competitive management system compared to conventional dairy production, with benefits that include less labor for animal care, lower investments in milking and housing, with similar income over feed cost [2]. In grazing dairy herds, superior forage nutritive value of pastures and increased pasture intake of cows is crucial for maintaining the production and health of the animals.

Pasture is the primary source of forage for organic dairies, and organic dairy cattle are required to have at least 120 d of grazing and 30% of their daily dry matter intake must come from pasture [3]. Although cattle need at least 120 d of grazing, cattle should be grazed more than 120 d based on the climate conditions in regions of the USA; and in the central region of the USA, grazing days may range from 120 to 200 d. In the central and northern United States, the pasture requirement is typically achieved by a May to October grazing season, and the economics of operations depends on pastures that provide a season-long supply of high-quality forage [4]. However, variation in weather may create a challenge for cool-season perennial (CSP) grasses, such as Kentucky bluegrass (*Poa pratensis* L.) and smooth bromegrass (*Bromus inermis Leyss. subsp. inermis*), and legumes such as white clover (*Trifolium repens* L.), which undergo a “summer slump” in production [4], and have slow growth in late autumn. Increasing forage quantity from pasture may reduce the costs of feeding harvested forage [5]. To create an extended forage supply, Ball et al. [5] recommended diversifying a pasture system to include warm-season grasses in the summer and cool-season grasses and legumes in the fall. However, these recommendations have never been evaluated under organic grazing conditions, and therefore, this was the overall objective of this research project.

Compared to monocultures, forage diversity may supply potentially more uniform pasture forage production during the growing season and improve soil health [6,7,8,9]. Pasture diversity may be increased by using grasses and forbs and by increasing numbers of grass and legumes species within species mixes [10]. Although legumes supply nitrogen to grasses and provide more energy in feedstuffs than grasses, legumes are generally less persistent and require improved levels of soil fertility [11]. Increased diversity in a farm’s forage production may be achieved by incorporating mixtures in individual pastures and by grazing separate pastures with different species [12].

Another approach to increasing diversity in a dairy farm’s pastures is to combine warm-season annual and perennial crops in separate fields [4]. Pasture-based dairy farms could use cool-season grasses and legumes for forage in the spring and early autumn, and warm-season (WSA) grasses, such as teff *(Eragrostis* tef) [13] and sudangrass (*Sorghum bicolor (L.) Moench subsp. drummondii (Steud.*) de Wet ex Davidse) [14], for forage in the summer. For extending the grazing season, cool-season annuals (CSA), such as small grains (e.g., oat (*Avena sativa L.*)) and brassicas (e.g., turnip (*Brassica rapa L*.)), have been proposed for the autumn [4,5,15]. Grazing producers in the central region of the USA have incorporated forage sorghum (*Sorghum bicolor (L.) Moench subsp. bicolor*), sudangrass, and sorghum x sudangrass hybrids into grazing systems [14]. Sorghum and sudangrass are desired for high yield during mid to late summer when cool-season grasses may be dormant [16]. Furthermore, teff has been proposed for grazing dairy farms; however, very few farmers use teff in grazing pastures. Teff has lower yield than sorghum-sudangrass [16,17], but there is little information on how dairy cattle may perform and graze on teff. No research has compared brown-midrib sorghum × sudangrass (BMRSS) and teff by grazing dairy cattle. Furthermore, the benefits of adding teff to a pasture production system has not been determined in northern and central regions of the United States.

In general, for the United States, total organic sales for dairy products and agronomic and vegetable crops increased by 31% from 2016. The number of certified organic farms increased by 17%, and the certified organic land increased by 9% [18]. Minnesota ranks ninth for the number of organic farms in the United States, with 635 farms, and the average organic farm is 110 hectares. There are 127 organic dairy farms in Minnesota, with 11,000 cows. Milk from dairy cows had the greatest value of sales ($38.2 million dollars) in 2019 [18,19].

Organic and grazing dairy producers in the United States, as well as around the world, are constantly searching for ways to improve milk production in their herds and have started to utilize WSA in pastures. The opportunity to potentially produce higher amounts of high-quality forage on the same amount of land may help organic farmers to achieve this goal of higher milk production [18]. It is important to determine high-quality forages that are intended for grazing in large-scale grazing systems. Therefore, the objective of this study was to compare pasture species used in a rotational grazing system in an organic dairy herd to evaluate whether incorporating warm-season grasses may be advantageous in a grazing system for organic dairy cows. Milk production and components, body weights, body condition score (BCS), and activity and rumination were evaluated for these two distinct grazing systems.

## 2. Materials and Methods

### 2.1. Experimental Design and Grazing Management

The organic dairy research herd at the University of Minnesota West Central Research and Outreach Center in Morris, MN, USA (45.59° N, 95.91° W) has a rotational grazing system, with 150 certified organic lactating dairy cows. The soil type at the research farm was a loamy soil with an average pH, N, P, K, and S of 7.25, 13 mg kg^−1^, 16 mg kg^−1^, 285 mg kg^−1^, and 9 mg^−1^, respectively. The certified organic production system is regulated by the United States Department of Agriculture National Organic Program [3]. The dairy cows of the herd were milked in a swing-9 milking parlor at 06:00 and 17:00 h. All procedures involving animal care and management were approved by the University of Minnesota Institutional Animal Care and Use Committee (#1508–32966A).

Two dairy grazing systems were compared. System 1 was a grazing system with a mixture of CSP forbs, grasses, and legumes (chicory (*Cichorium intybus* L.), meadow bromegrass (*Bromus riparius Rehmann*), meadow fescue (*Schedonorus pratensis* (Huds.) P. Beauv.), orchardgrass (*Dactylis glomerata* L.), perennial ryegrass (*Lolium perenne* L.), alfalfa (*Medicago sativa* L.), red clover *(Trifolium pratense* L.), and white clover (*T. repens* L.)). The CSP were established with a no-till drill and planted into existing pastures of smooth bromegrass in April 2012. Pure live seeding rates for System 1 were 1.2 kg/ha for chicory, 2.3 kg/ha for meadow bromegrass, 9 kg/ha for meadow fescue, 3.4 kg/ha for orchardgrass, 4.5 kg/ha for perennial ryegrass, 4.5 kg/ha for alfalfa, 3.4 kg/has for red clover, and 4.5 kg/ha for white clover. The CSP pastures were not fertilized and no manure was applied. Pasture System 2 was a combination of System 1 and WSA grasses (brown-midrib sorghum-sudangrass (*Sorghum bicolor (L.) Moench subsp. drummondii (Steud.)* and teff) and were grazed during July, August, and September. The BMRSS and teff were planted into separate monoculture pastures, and CSA were planted together. In October, CSA were included in the pasture rotation and cows only grazed CSA twice during each year of the grazing season. During the late summer, a 0.80 h section of System 2 pastures of the BMRSS and teff were plowed and CSA were planted into prepared seedbeds. This was not additional land for growing oat and turnips. Oat and turnips were seeded at a rate of 112 and 7 kg/ha, respectively, within the CSA mixture. Both species were combined and seeded together.

A total of 46.4 ha of pasture land was used for this study. System 1 had 17.4 ha of CSP pasture across five separate pastures, and System 2 had 11.9 ha of CSP pastures across four separate pastures and 17.1 ha of WSA across three separate pastures. This study evaluated CSP and WSA across 12 specific pastures, and individual pastures were divided into replicated paddocks for grazing of lactating dairy cattle. Replicated paddocks within a pasture were approximately 0.60 ha to implement rotational grazing. The paddocks were replicated three times across the two pasture systems. System 2 had more land allocated to pasture to account for the rotation of BMRSS and teff grass grown, as well as the incorporation of CSA in the rotation grazing system. Each of the 3 replicated groups of cows that grazed System 2 had an equal amount of land for CSP and WSA. Cows grazed all of the land and no pastures were made into silage or baleage.

For the entire study, pasture experimental units were grazed with rotations within pastures. The experimental design of this study was similar to methods outlined by Fisher [20] for grazing research trials that included animal groups on pastures. Grazing of CSP started when forages in the paddocks was a height of 20 to 30 cm tall and paddocks were sized to leave 7 to 13 cm of residual grass stubble. The pre- and post-grazing CSP height was normal for the specific CSP grown in the Upper Midwest of the United States. The rotation length of CSP pastures averaged 30 d. Pastures were not fertilized.

The WSA grasses were seeded in May 2014 and 2015. The WSA were seeded into pastures that were previously Kentucky bluegrass and smooth bromegrass. The seeding rates for BMRSS and teff were 22 and 9 kg/ha, respectively. The BMRSS and teff were seeded 2.5 to 4 cm in depth when soil temperatures reached 16 °C. The BMRSS was at least 45 cm in height before cattle grazed to minimize the risk of prussic acid poisoning. Teff was grazed from 20 to 30 cm, with 7 to 13 cm of stubble. The rest time for each paddock withing a pasture varied from 21 to 35 d depending on weather and forage biomass. The WSA pastures were not fertilized and no manure was applied.

Daily weather was from the University of Minnesota West Central and Outreach Center Weather station in Morris, MN. The monthly high, low, and average temperature and total monthly precipitation for the six months of each year of this study during the grazing season are in Table 1. Minnesota has a variety of weather conditions and is a humid continental climate with warm and humid days and cool evenings. Typically, spring has many thunderstorms that provide moisture, summer is hot and humid, and the autumn typically is much cooler with lower precipitation.

### 2.2. Grazing of Dairy Cattle

Ninety lactating organic dairy cows grazed two pasture systems (six groups of 15 cows each) over two summer grazing seasons (2 June 2014 to 22 October 2014; 18 May 2015 to 17 October 2015). Cows were balanced within the three replicates by breed (Holstein and crossbred), days in milk (mean = 137 days and ranged 5 to 260 days), and parity (parity ranged 1 to 7), and randomly assigned to System 1 or System 2. Average body weights of cows were 499 kg. All grazing groups of lactating cows were provided with free choice mineral and 2.54 kg/DM/cow/day ground corn.

Cows were managed in an intensive rotational grazing method for both systems. Cows grazed 22 h per day, except for periods when cows were milked twice per day. The cows in System 1 always moved to a new paddock within CSP pasture. For System 2, cows were moved to a new CSP paddock or to a WSA paddock if BMRSS or teff could be grazed. Generally, the cows moved to a new paddock of fresh grass every 2 days. In October, CSA pastures were included in the rotation so the System 2 cows could possibly be moved to either CSP, a WSA grass, or CSA. Stocking density was 0.387 hectare per cow for System 1 and 0.644 hectare per cow for System 2. Forage clippings were taken within 48 h prior to cows being let into a new paddock to estimate pasture dry matter allowance and forage nutritive value. Grazing height and forage availability were measured before cows moved into a new paddock and after cows left a paddock with a Jenquip pasture plate meter (Jenquip, Feilding, New Zealand). The pasture meter was calibrated in early May and used the equation Y = 122X + 955, where Y was the cover reading (DM/ha) and X was the height of the grass in cm to determine the DM/ha available in the pasture. The equation reported was entered into the pasture plate meter and the equation was based on the 0.5 cm increments as suggested by the manufacturer.

### 2.3. Milk Production, BW, BCS, Activity and Rumination

Daily milk production from individual cows in each year was measured with Boumatic Smart Dairy system (Madison, WI, USA) and bi-weekly measures of fat percentage, protein percentage, MUN, and SCS. Milk samples were analyzed by Stearns DHIA Laboratories (Sauk Centre, MN, USA) with a 4000/5000 Combi-Foss Milk Analyzer (Hilleroed, Denmark). A Skalar Analyzer (Breda, Netherlands) determined MUN. To evaluate animal health, cow body weight was recorded bi-weekly using a digital scale. As cows exited the milking parlor, BCS was rated by the same person who had recorded BCS of cows for many research studies. Body condition scores were 1 = excessively thin to 5 = excessively fat and were recorded by the same person that has recorded BCS of cows for many research studies. For analysis, production and health measures were averaged across cows and appropriate dates in each group.

All cows had an activity and rumination collar (SCR Engineers Ltd., Netanya, Israel) around their the neck [20]. Activity of cows was daily activity units. Rumination of cows was measured in minutes per day [21]. When cows were moved to the milking parlor for milking, data were collected from cows via a receiver. The raw data were transmitted to the computer in the farm office and processed through the SCR DataFlow II software (Data Flow Software; SCR Engineers Ltd., Netanya, Israel). Rumination results are presented in min/day and activity results are presented as SCR units based on a proprietary algorithm.

### 2.4. Statistical Analysis

All response variables were analyzed in relation to fixed effects of grazing system (System 1 or System 2), forage type (CSP, BMRSS, teff, CSA) nested within system, year (2014 or 2015), and repeated measures over dates within system-by-year combinations, as appropriate. Replicates nested within combinations of system and year were treated as a random effect. The compound symmetry covariance over dates was used because it had the lowest Akaike’s information criterion [22]. PROC MIXED of SAS [23] analyzed fat percentage, protein percentage, MUN, SCS, body weight, and BCS, each averaged over cows within each group. PROC HPMIXED of SAS was used for average daily milk production, daily rumination, and daily activity. All observations within replicates and dates were averaged for analyses. Replicate pen of cows on pasture was the experimental unit for analysis. All results were reported as least squares means, with significance declared at *p* < 0.05.

## 3. Results

### 3.1. Weather

Weather data recorded during the 6 month grazing seasons of 2014 and 2015 are summarized in Table 1. The first year was cooler and drier than the second year. Average temperature was 16 °C in 2014 and 17.1 °C in 2015. Seasonal total precipitation was 52.4 cm in 2014 and 57.1 cm in 2015 (Table 1). In both years, May and October were cooler than the months of June to September. Months with the highest precipitation across the two years include June 2014, August 2014, May 2015, and August 2015 (Table 1). Lower total precipitation occurred in 2014 compared to 2015. The driest months were September 2014 and 2015 and October 2014 (Table 1). The weather conditions for two years of this study provided the insight to study the CSP and WSA pastures under different growing conditions.

### 3.2. Milk Production between Systems

Least squares means and standard errors of means for milk production, SCS, and MUN for season-long grazing of dairy cows in System 1 and System 2 are in Table 2. Cows from both System 1 and System 2 grazing systems had similar milk production, fat percentage, protein percentage, SCS, and MUN (Table 2).

Milk production varied among specific forages within the two pasture systems (Table 3). Milk production was greater for cows grazing BMRSS (15.4 kg d^−1^) compared to CSP (14.4 kg d^−1^) and teff grass (14.5 kg d^−1^) within System 2. However, cows grazing CSP for the entire grazing season in System 1 had similar milk production to cows on all forages in System 2 (Table 3). Responses to teff grass or to the CSA mixture were not significant. The MUN was lower for cows on BMRSS than cows grazing CSP (Table 3). In contrast, levels of fat, protein and SCS did not differ between System 1 and System 2 groups (Table 2), nor among System 2 groups when they were grazing in their different paddocks (Table 3).

**Figure 1:**
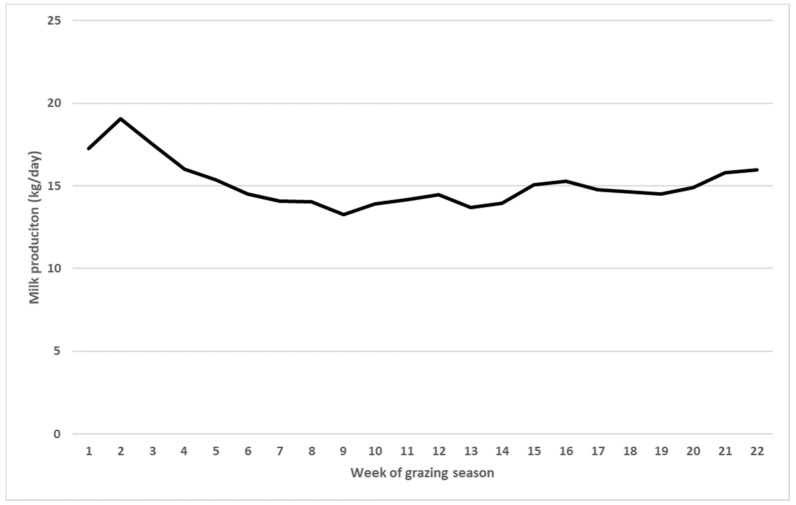
Milk production (kg d^−1^) across the years and weeks of the grazing season for both System 1 and System 2 cows. Values are the least squares means of two years of all the 90 cows in this study across the 22 weeks of the grazing season.

Milk production across the grazing season for both System 1 and System 2 increased rapidly when cows were moved from winter confinement to pastures. During the summer, when temperatures increased, cows experience a decrease in milk production, and then, milk production gradually increased towards the end of the grazing season (Figure 1).

### 3.3. Body Weight, Body Condition Score, Activity, and Rumination

Least squares means and standard errors for rumination, activity, body weight, and BCS for cows in System 1 and System 2 are shown in Table 4. Cows had similar BCS (3.1) and body weight throughout this study, whether they were in System 1 or System 2. Cows in System 1 and System 2 had similar activity levels across the grazing season. Rumination (470 min/day) was lower for cows grazing System 2, which incorporated the WSA, than cows in System 1 (530 min/day) (Table 4). Furthermore, cows grazing CSP in System 2 had lower rumination than cows grazing CSP in System 1 (Table 4).

Cows had similar BW and BCS on all types of forages (Table 5). Cows grazing teff grass had similar activity to cows grazing CSP pastures in either system. Cows had lower rumination when grazing BMRSS and teff grass than when grazing CSP (Table 5).

## 4. Discussion

In a companion paper, Ritz et al. [24] reported forage yields that were greater for forages in System 2 compared to System 1. Furthermore, crude protein (CP) was greater for CSP and acid detergent fiber (ADF) and neutral detergent fiber (NDF) were similar for forage species across the grazing season. However, total tract NDF digestibility differed by month across the grazing study. Net energy for lactation was greater for CSP (5.61 MJ/kg) compared to WSA grasses (5.43 MJ/kg). Forage nutritive value decreased from June and July and was the lowest during September and October. The results of the study [24] suggested that climatic conditions and precipitation may play a large factor in the forage nutritive value of cool-season and warm-season grasses in an organic pasture production system.

Cows in both systems followed similar trends in milk production across the grazing season, based on the weather patterns. When there were extreme weather events such as high temperature that affected cow health, cows in both pasture systems experienced a slump in milk production (Figure 1; Table 1). Although forage yield and nutritive value are important for production, production for cows in grazing systems is also highly associated with weather patterns [25].

The similar milk production of cows in System 1 and System 2 may be because cows in both systems produced relatively low levels of milk (average of 15 kg d^−1^) due to the low energy values of the forage-based diets [24]. The cows in each pasture system were supplemented with only 2.54 kg/DM/day of grain. In another study with cows from the same organic herd that grazed CSP without supplementation, Sjostrom et al. [26] reported similar production levels for cows consuming 100% pasture and no grain compared to those that were supplemented with minimal amounts of grain. Many studies that have compared organic dairy production to conventional production noted a lower average daily milk production in organic farms [1,27,28,29]. However, when animal requirements are satisfied, milk production results were not different between organic and conventional systems [30]. Therefore, the breed composition of cattle on particular farms may influence milk production results more than the organic farming system [31,32]. Additionally, milk production may be lower for this study because cows at the research center may typically walk a greater distance to the milking parlor for milking [26]. Other factors that may explain for the lower milk production of the cows in this herd are: the cows were on average mid-lactation cows (mean = 137 day in milk), which would have lower milk production than early lactation cows; increased fly pressure during the day may lower dry matter intake by cows [26] and, thus, lowering milk production; and cows may experience heat stress during the day because of reduced access to shade in pastures [33]. High MUN levels may have also had an effect on milk yield of the cows in this study. Further research is needed to determine effects for cows in different types of organic systems when utilizing these WSA in a grazing system, for example for organic farms that may include higher levels of concentrate or supplement pasture with TMR or farms with different breeds of cows.

One factor that may have contributed to the higher milk production of cows grazing BMRSS is the lower MUN in those cows [34,35]. Converting ammonia to urea in the liver is 12 Kcal/g excess N excreted for cows [36], thereby depleting the amount of energy available for milk production. The MUN was above normal levels of 7 to 9 mg dL^−1^ [35] the entire grazing season and was not at or below those levels. However, research regarding the relationship between MUN and production is inconclusive, as some studies show positive relationships [37], no relationship [38] or negative relationships [34,35]. The lower MUN of cows grazing BMRSS may be due to the lower crude protein content of BMRSS compared with CSP [24], as MUN levels are associated to levels of dietary CP [39].

Roche et al. [40] determined that the optimum milk production would come from cows that have a BCS of 3.5, but no decrease in milk production of cows was reported at a BCS of 3.0. Those results correspond to the results of the current study for BCS of 3.1 and 3.0 throughout the grazing season. Milk production may be correlated with BCS loss [40], but there was no change in BCS and no difference in milk production between cows in the different systems in the current study. Differences in body weight were not observed between System 1 and System 2 cows and the standard errors of means for cows grazing teff and CSA were greater than cows grazing CSP and BMRSS. The higher standard errors are more than likely caused by small number of observations for body weight when cows were grazing teff or CSA. Therefore, more observations of body weight are needed in additional studies for these particular forages to determine whether body weight had an effect on cows grazing teff or CSA.

Rumination is considered a significant component of digestion and animal well-being for ruminants [41]. Lower rumination times may be caused by dietary and animal factors including lower NDF, higher digestibility, lower dry matter intake, and may decrease milk production or lead to a greater risk of acidosis [42]. Cows in both systems had rumination times that were within the range of 428–555 min/day [42,43,44]. Cows in System 2 were grazing WSA that was consistently at a lower maturity than grass in System 1 throughout the grazing season due to the different growing patterns of the different CSP [24]. Quite possibly, this may have led to these cows having lower amounts of physically effective fiber in their diet than cows in System 1, which may have experienced increasing maturity of CSP as the grazing season progressed [45]. Ritz et al. [24] reported NDF was greater for WSA grasses during the fall compared to CSP. Depending on climatic conditions of month and year, the ADF was greater for WSA compared to CSP for cows in the current grazing study [24]. Furthermore, in a continuous culture system with CSP and WSA from the current study, NDF and ADF were numerically greater for BMRSS compared to CSP [46].

As grazing dairy cows reduce rumination to increase effective grazing [47], it may have been possible that cows grazing BMRSS and teff in the current study were spending more time eating than ruminating, which is supported by the higher activity levels observed for cows grazing BMRSS. Furthermore, cows grazing CSP in System 2 had lower rumination than cows grazing CSP in System 1. Activity was highest for cows grazing BMRSS (Table 5), which was probably due to the physical structure of the plant, as cows must be more active to move about the pasture because of the tall stems and long leaves of the BMRSS. Activity was lowest for cows in CSA (Table 5), which could be due to the cooler air temperatures during October when cows were grazing these CSA pastures, as cooler temperatures maybe be associated with lower activity levels [48].

Organic farmers may readily comply with the organic pasture rule by incorporating WSA into a grazing system to extend the grazing season and increase forage yield of grasses during a summer grazing season. Therefore, WSA in dairy grazing systems may be beneficial to compensate for temperature and precipitation that influences pasture forage yield. Milk components and production may also be maintained when WSA are incorporated in a grazing system for organic dairy cattle. Pasture management and forage species selection may influence the forage nutritive value of pasture for grazing dairy cattle.

A disadvantage of WSA in a grazing system is that they may not be ready for grazing until the middle of the grazing season during the hot months of the summer. However, incorporating WSA and CSA into a pasture production system may produce more forage yield across the entire grazing season. Profitability analysis of WSA and CSA for pasture production systems is need and there may be a higher cost for seed and additional labor to incorporate WSA and CSA into a pasture. However, there may be a greater benefit to including WSA in a grazing rotation, and the tradeoffs between the amount of land needed for WSA and forage yield milk production must be explored. Additional pasture land may be needed on a grazing farm to account for the addition of WSA and CSA into the rotation. Further research should be conducted on the economics of incorporating WSA grasses into milk cow grazing systems, and on the optimal proportion of WSA grasses to minimize effects of summer slump with CSP, to extend the autumn grazing season, and to minimize economic risk associated with seed costs, under a wider range of weather effects.

## 5. Conclusions

Organic and conventional graziers have expressed concerned that warm-season grasses may be detrimental to cow productivity, health and activity. Low to moderately yielding cows with low supplementation levels that grazed BMRSS produced more milk than cows grazing more cool-season perennial mixtures. Additionally, cows that grazed warm-season grasses had lower MUN levels and this would be considered an advantage to assist in lowering overall MUN for all cows in grazing dairy herd. Body weight and BCS were not affected by forage treatment. Cows that grazed perennial grasses had lower rumination than cows that grazed warm-season annuals. Warm-season annual grasses can successfully be incorporated into grazing systems in the Upper Midwest USA and similar environments globally. Depending on climatic conditions around the world, grazing dairy producers internationally may use warm-season grasses to increase pasture growth and provide more forage to grazing dairy cattle.

## Figures and Tables

**Table 1:** Monthly average temperatures and precipitations during the 2014–2015 grazing season at the West Central Research and Outreach Center, Morris, MN, USA.

Year	Temperature (°C)				Rainfall (cm)
	Month	High	Low	Average	Monthly Total
2014	May	17.2	6.7	12.8	7.5
	June	24.4	13.9	19.4	20.8
	July	25.6	13.9	20.0	4.6
	August	25.0	15.0	20.0	14.6
	September	21.7	9.4	15.6	3.6
	October	15.0	1.7	8.3	1.3
2015	May	18.9	7.2	13.3	19.7
	June	25.6	13.9	20.0	4.7
	July	27.2	16.1	21.7	9.3
	August	25.6	13.3	19.4	16.0
	September	25.0	12.2	18.9	3.4
	October	15.6	3.3	9.4	4.0

**Table 2:** Least squares means and standard errors of two years for milk production, milk components, somatic cell score, and milk urea nitrogen for organic dairy cows in two grazing systems. Values are the least squares means of two years, three replicates within each year, 15 cows per replicate.

Variable ^1^	System 1 Grazing System		System 2 Grazing System	
	Mean	SEM	Mean	SEM
Milk production (kg d^−1^)	14.6	0.53	14.8	0.54
Milk urea nitrogen (mg dL^−1^)	12.5	0.8	11.5	0.9
Fat percentage (%)	3.9	0.1	3.8	0.1
Protein percentage (%)	3.2	0.02	3.2	0.02
Somatic cell score	4.1	0.1	4.1	0.1

^1^ No significant difference pasture grazing systems for all variables. System 1 was cool-season perennials and System 2 was cool-season perennials with warm-season annual grasses and cool-season annuals.

**Table 3:** Least squares means and standard errors for milk production, milk components, and SCS for 2 years, for periods when Systems 2 cows were grazing in specific paddocks within the System 2 forage system. Values are the least squares means of two years, three replicates within each year, 15 cows per replicate.

Variable	System 1 Grazing System		System 2 Grazing System							
	CSP Species		CSP Species		BMR Sorghum-Sudangrass		Teff		CSA Annuals	
	Mean	SEM	Mean	SEM	Mean	SEM	Mean	SEM	Mean	SEM
Milk (kg d^−1^)	14.6 ^a,b^	0.5	14.4 ^b^	0.5	15.4 ^a^	0.6	14.5 ^b^	0.6	14.9 ^a,b^	0.6
MUN (mg dL^−1^)	12.5 ^a^	0.8	13.3 ^a^	1.0	9.3 ^b^	1.3	10.5 ^a,b^	2.6	12.5 ^a,b^	2.4
Fat (%)	3.9	0.6	3.8	0.1	3.8	0.2	3.8	0.4	3.8	0.3
Protein (%)	3.2	0.02	3.2	0.03	3.2	0.04	3.1	0.08	3.2	0.08
Somatic cell score	4.1	0.1	4.0	0.1	4.1	0.1	4.5	0.2	3.7	0.2

^a,b^ Means within a row with different superscripts are different at *p* < 0.05. System 1 was cool-season perennials (CSP) and System 2 was CSP with warm-season annual grasses (WSA) and cool-season annuals (CSA) of oat and turnip.

**Table 4:** Least squares means and standard errors for measures of body weight, body condition score (BCS), level of general activity, and rumination time, averaged across the 2 years of the grazing seasons for cows in System 1 and System 2. Values are the least squares means of two years, three replicates within each year, 15 cows per replicate.

Variable	System 1 Grazing System		System 2 Grazing System	
	Mean	SEM	Mean	SEM
Body weight (kg)	481	10.2	494	12.4
Body condition score	3.1	0.04	3.1	0.04
Activity (SCR units)	596	34.5	614	34.9
Rumination (min/day)	530 ^a^	4.4	470 ^b^	5.1

^a,b^ Means within a row with different superscripts are different at *p* < 0.05. System 1 was cool-season perennials and System 2 was cool-season perennials with warm-season annual grasses and cool-season annuals.

**Table 5:** Least squares means and standard errors for body weights, BCS, measures of general activity, and rumination times, averaged across two grazing years for organic cows in System 1 and System 2. Values are the least squares means of two years, three replicates within each year, 15 cows per replicate.

Variable	System 1 Grazing System		System 2 Grazing System							
	CSP Species		CSP Species		BMR Sorghum-Sudangrass		Teff		CSA Annual	
	Mean	SEM	Mean	SEM	Mean	SEM	Mean	SEM	Mean	SEM
Body weight (kg)	481	10.2	491	12.3	494	17.8	498	25.6	492	36.3
Body condition score	3.1	0.04	3.1	0.04	3.1	0.05	3.0	0.06	3.1	0.08
Activity (SCR units)	596 ^b^	34.5	595 ^b^	34.8	663 ^a^	35.5	615 ^b^	38.4	582 ^c^	41.4
Rumination (min/day)	530 ^a^	4.4	506 ^b^	4.9	445 ^c^	5.9	437 ^c^	9.1	493 ^a,b^	11.7

^a,b,c^ Means within a row with different superscripts are different at *p* < 0.05. System 1 was cool-season perennials (CSP) and System 2 was CSP with warm-season annual grasses (WSA) and cool-season annuals (CSA) of oat and turnip.

## Data Availability

The data presented in this study are available on request from the corresponding author.
